# Retraction: Opportunities and challenges of disease biomarkers: a new section in the journal of translational medicine

**DOI:** 10.1186/1479-5876-11-144

**Published:** 2013-06-11

**Authors:** Xiangdong Wang, Peter A Ward

**Affiliations:** 1 Department of Respiratory Medicine, Biomedical Research Center, Zhongshan Hospital Qing-Pu Branch, Fudan University, Shanghai, China; 2 Clinical Bioinformatics, Lund University Hospital, Lund, Sweden; 3 Department of Pathology, University of Michigan Medical School, Ann Arbor, MI, USA

## 

The publisher has retracted this article [1] because it was republished in error [2] due to technical reasons. BioMed Central apologize to the authors and readers for the error and any inconvenience caused.
